# OmpA Protein-Deficient *Acinetobacter baumannii* Outer Membrane Vesicles Trigger Reduced Inflammatory Response

**DOI:** 10.3390/pathogens10040407

**Published:** 2021-03-31

**Authors:** Jūratė Skerniškytė, Emilija Karazijaitė, Asta Lučiūnaitė, Edita Sužiedėlienė

**Affiliations:** 1 Institute of Biosciences, Life Sciences Center, Vilnius University, Saulėtekio ave. 7, LT-10257 Vilnius, Lithuania; e.karazijaite@gmail.com; 2 UPR 9002, Architecture et Réactivité de l’ARN, Université de Strasbourg, CNRS, F-67000 Strasbourg, France; 3 Institute of Biotechnology, Life Sciences Center, Vilnius University, Saulėtekio ave. 7, LT-10257 Vilnius, Lithuania; asta.luciunaite@bti.vu.lt

**Keywords:** *Acinetobacter baumannii*, outer membrane vesicles, macrophages, inflammation, inflammasome

## Abstract

Multidrug resistant *Acinetobacter baumannii* shows a growing number of nosocomial infections worldwide during the last decade. The outer membrane vesicles (OMVs) produced by this bacterium draw increasing attention as a possible treatment target. OMVs have been implicated in the reduction of antibiotic level in the surrounding environment, transfer of virulence factors into the host cells, and induction of inflammatory response. Although the evidence on the involvement of OMVs in *A. baumannii* pathogenesis is currently growing, their role during inflammation is insufficiently explored. It is likely that bacteria, by secreting OMVs, can expand the area of their exposure and prepare surrounding matrix for infection. Here, we investigated the impact of *A. baumannii* OMVs on activation of macrophages in vitro. We show that OmpA protein present in *A. baumannii* OMVs substantially contributes to the proinflammatory response in J774 murine macrophages and to the cell death in both lung epithelium cells and macrophages. The loss of OmpA protein in OMVs, obtained from *A. baumannii* ∆*ompA* mutant, resulted in the altered expression of genes coding for IL-6, NLRP3 and IL-1β proinflammatory molecules in macrophages in vitro. These results imply that OmpA protein in bacterial OMVs could trigger a more intense proinflammatory response.

## 1. Introduction

Opportunistic bacterium *Acinetobacter baumannii* is responsible for numerous cases of hospital-related infections worldwide [1]. The ability to persist on medical devices and survive in the patients with suppressed immunity rendered this bacterium to one of the most successful colonizers in medical units. The successful spread in the infected organism is mainly due to combined effect of various virulence-related factors, among which the outer membrane protein A (OmpA) is studied in detail and is considered as one of the key virulence factors of *A. baumannii* [2]. OmpA porin is composed of membrane β-barrel and periplasmic domains. While periplasmic domain interacts with peptidoglycan and is responsible for the stability of bacterial outer membrane, the β-barrel domain transfers some β-lactams, such as sulbactam and imipenem into bacterium [3]. The role of OmpA in biofilm formation, adhesion to abiotic surfaces and to the cells of various lineages, also resistance to the host immune system has been demonstrated [4]. Due to the high impact on *A. baumannii* pathogenesis, OmpA protein is being considered as a promising treatment target and vaccine candidate [4].

The colocalization of OmpA in the mitochondria and nucleus has been observed in infected cells in vitro [5,6] raising a question of how OmpA protein is being transported into the target cell. One possible explanation could be outer membrane vesicles (OMVs) secreted by bacteria [7,8]. Recently, it was demonstrated that OmpA present in *A. baumannii* OMVs is able to induce mitochondrial fragmentation in lung epithelium cells [9].

Bacterial OMVs are spherical structures of approximately 20–300 nm in diameter, which detach from the outer membrane encapsulating the components of periplasmic space, also inner-membrane and some cytoplasmic proteins [10]. These structures have been highlighted when it was observed that bacteria might transfer virulence factors, modulate immune system and inactivate antibiotics extracellularly by secreting OMVs [11,12,13]. OMVs are secreted by various Gram-negative and Gram-positive bacteria, thereby implying their role in the bacterial physiology [10,14].

So far, little is known about the involvement of OMVs and their components in *A. baumannii* pathogenesis. OMVs isolated from multidrug resistant *Escherichia coli* were able to inactivate β-lactam antibiotics [15]. Resistant bacteria “pack” antibiotic-inactivating enzymes e.g., β-lactamases in OMVs [16]. After the transfer of selective antibiotic into the OMVs, relevant enzymes hydrolyze it. Recently, we have demonstrated that OMVs isolated from multidrug-resistant *A. baumannii* strain inactivated ampicillin in the incubation medium [17]. The cytotoxic and immunomodulatory properties of OMVs secreted by *A. baumannii* have been also observed [7,9,18]. However, the exact mechanisms of the interplay between *A. baumannii* OMVs components and the host immune system remain obscure.

In this study we aimed to investigate the cytotoxic and immunomodulatory properties of OMVs secreted by clinical *A. baumannii* strain and its *ompA* gene deletion mutant. We demonstrated that OmpA protein could modulate the response of murine macrophages not only by being incorporated into the outer membrane of bacteria [19], but also as the component of OMVs, thereby expanding its effect in the infected host tissues.

## 2. Results and Discussion

### 2.1. OmpA Protein-Deficient A. baumannii OMVs Show Reduced Ability to Induce Cell Death

**Figure 1:**
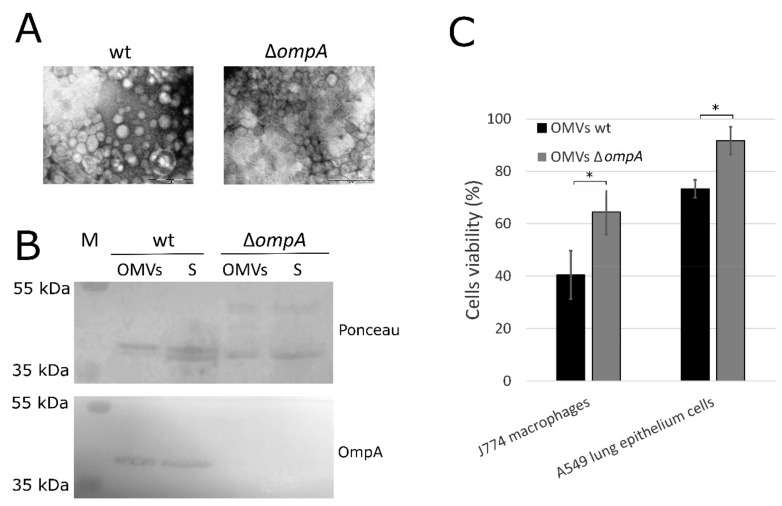
Outer membrane vesicles (OMVs) from *Acinetobacter baumannii* wt strain and its ∆*ompA* mutant. (**A**) OMVs isolated from *A. baumannii* wt strain and its *ompA* deletion mutant were visualized by TEM (scale bar 0.2 µm); (**B**) Western blot analysis of OmpA protein (38 kDa) in precipitant (OMVs) and supernatant (S) fractions after ultracentrifugation of filtered growth medium. Nitrocellulose membrane was stained with Ponceau Rouge stain. The serum from mice, immunized with OmpA protein, was used as primary antibodies. M—protein marker. Membrane was developed with Pierce one-step ultra TMB blotting solution (Thermo Fisher Scientific, Walkersville, MD, USA); (**C**) cell viability assay assessed by MTT method. J774 mice macrophages and A549 human epithelium cells were incubated for 24 h with OMVs produced by *A. baumannii* wt strain and its *ompA* deletion mutant. The viability of cells incubated with PBS was considered as 100% and was compared to the viability of cells exposed to OMVs. Error bars represent standard deviations of three independent experiments. The significance was assessed by t-test (* *p* < 0.05).

*A. baumannii* clinical strain Ab_169_ belonging to international clone I (IC I) ST231 and its *ompA* deletion mutant [20] were used for the isolation of OMVs. OMVs were purified by ultracentrifugation and the fractions were visualized by transmission electron microscopy (TEM) (Figure 1A). Mutant strain produced approximately three times more OMVs compared to wt strain. The proteins were fractionated by SDS-PAGE (Figure S1) and the presence of OmpA protein in OMVs was confirmed by Western-blot analysis (Figure 1B).

The cytotoxicity of OMVs secreted by *A. baumannii* wt strain and its *ompA* deletion mutant to J774 mice macrophages and A549 human lung epithelium cells was investigated. Cells were incubated with OMVs for 24 h and cell viability was assessed as described in Section 3. OMVs produced by *A. baumannii* wt strain reduced viability of J774 macrophages and A549 cells to approximately 40% and 73%, respectively, compared to PBS-treated control. Accordingly, approximately 65% of J774 macrophages and 92% of A549 cells survived after incubation with OMVs obtained from *A. baumannii ompA* deletion mutant (Figure 1C). Therefore, OmpA-deficient OMVs demonstrated reduced cytotoxicity compared to OMVs from wt strain both for macrophages and lung epithelium cells. The unequal cytotoxicity of *A. baumannii* OMVs produced by two different clinical *A. baumannii* strains to mice macrophages RAW264.7 and A549 cells has been demonstrated [21]. In one of the early investigations, OMVs obtained from the OmpA-deficient *A. baumannii* 19606∆*ompA* strain did not induce cell death in human monocytes U937 [7]. Several studies identified OmpA protein as a virulence factor of *A. baumannii*, which is involved in biofilm formation, adhesion to eukaryotic cells and host colonization [22,23,24]. Our results show that *A. baumannii* OmpA protein being incorporated into OMVs contributes to their cytotoxicity. Macrophages were more susceptible to OMVs compared to lung epithelium cells. As macrophages are professional phagocytes, they are assigned to induce acute inflammatory response to the infection. These results are in agreement with another study where RAW264.7 macrophages were also more susceptible to OMVs exposure compared to A549 cells [21].

### 2.2. OmpA-Deficient OMVs Elicit Reduced Inflammatory Response in Mice Macrophages In Vitro

**Figure 2:**
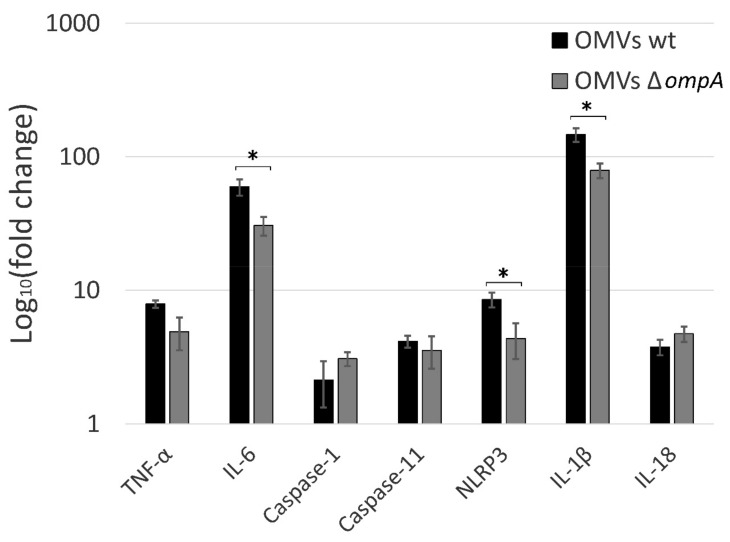
The expression of proinflammatory molecules in J774 mice macrophages after 24 h incubation with *A. baumannii* OMVs assessed by qPCR. Relative expression was compared to the macrophages incubated with PBS. Error bars represent standard errors of three independent experiments. The significance was assessed by t-test (* *p* < 0.05).

*A. baumannii* OMVs can induce immune response in immune cells [18,21]. In order to elucidate if the loss of OmpA protein in OMVs affects the inflammatory response in macrophages, we incubated J774 cells with OMVs, produced by *A. baumannii* wt strain and its *ompA* deletion mutant for 24 h. After incubation, cellular RNA was extracted, and gene expression was assessed by qPCR using proinflammatory molecules coding genes as targets. Results demonstrated the induction of all tested genes after treatment of macrophages with OMVs compared to PBS-treated control, showing that OMVs can act as powerful inductors of inflammation (Figure 2). Moreover, the reduction in the expression of a set of proinflammatory cytokines was observed when macrophages were incubated with OMVs produced by *ompA* deletion mutant compared to wt strain. Macrophages are the major producers of TNFα [25]. In the presence of infection, TNFα is a key regulator of inflammatory response through altering the production of proinflammatory cytokine cascades, activating phagocytosis, production of nitric oxide (NO) and, finally, induction of cell death. Our results demonstrated a moderate reduction of *Tnfα* gene expression when macrophages were incubated with OMVs derived from ∆*ompA* strain compared to wt strain (Figure 2). The trait that OMVs from distinct bacterial strains can induce different TNFα production level has been already observed [21] indicating that *A. baumannii* strains might regulate OMVs-mediated virulence through modulation of their proinflammatory properties. 

The macrophages produce IL-6 during the infection in order to activate other immune cells, such as neutrophils or B cells [25]. As can be seen in the data, presented in Figure 2, OmpA deficiency resulted in a significantly reduced ability of OMVs to induce *Il-6* gene expression, thereby indicating that OmpA present in OMVs acts as inducer of immune cells recruitment into infection site. It must be noted, however, that OmpA-deficient OMVs still were able to induce strong *Il-6* gene expression in murine macrophages, indicating that OMVs can recruit a wide immune response, possibly, due to the other pro-inflammatory molecules present in OMVs.

### 2.3. A. baumannii OMVs Trigger the Inflammasome Activation 

According to the recent studies *A. baumannii* could not only induce apoptosis in immune cells, but also pyroptotic cell death, in which activated gasdermin D forms pores in the cell plasma membrane [26,27]. We observed that *A. baumannii* OMVs triggered modest induction of caspase-1 expression in J774 macrophages (Figure 2). Caspase-1 is a key peptidase in pyroptosis, which is activated by formation of inflammasome, a protein complex of the innate immune system, composed of CARD/PYD domain containing proteins and Apoptosis associated Speck-like protein containing a CARD (ASC) adaptor proteins [28]. The inflammasome induces the cleavage of pro-caspase-1 into its active form. In the noncanonical inflammasome, caspase-11 is activated by direct binding to bacterial LPS leading to pyroptosis [28]. Interestingly, we show in this study, that the expression of both caspases was induced when macrophages were incubated with *A. baumannii* OMVs. These results are in agreement with the recent studies on the response of bone marrow-derived macrophages (BMDMs) during *A. baumannii* infection, where the importance of both caspase-1 and caspase-11 was demonstrated, suggesting that these two pathways complement each other [27,29]. 

There are different types of inflammasome complexes based on their inflammasome receptors. Pathogen-Associated Molecular Patterns (PAMPs) such as LPS, LOS, bacterial surface proteins, or TNFα activate NLRP3 inflammasome [30]. In addition to the formation of inflammasome complex, the priming step is needed. After sensing, the extracellular stimuli pattern recognition receptors (PRRs) activate MyD88/NF-κB pathway, resulting in the expression of *Nlrp3* and other proinflammatory genes [30]. Our study showed the increased expression of *Nlrp3*, *Il-1β* and *Il-18* genes in the presence of OMVs, implying the activation of MyD88/NF-kB signaling pathway and induction of proinflammatory cytokines in macrophages. Interestingly, OmpA-deficient OMVs elicited significantly reduced expression of *Nlp3* and *Il-1β,* but not *Il-18* gene (Figure 2). The decreased expression of proinflammatory proteins could be a result of the reduced production of TNFα, elicited by OmpA-deficient OMVs compared to wt OMVs, what could cause a weaker priming signaling through TNF receptor. 

The observed *Il-18* gene expression profile, distinct from that of *Nlrp3* and *Il-1β* genes, indicates that different induction pathway might be involved, possibly through the acceleration of different transcription factors. This is in agreement with other studies, where distinct induction pathways for IL-1β and IL-18 were observed [31]. Based on the current knowledge, the expression of *Nlrp3* and *Il-1β* genes might be induced via MyD88/NF-κB pathway through activation of TLR and TNF receptors [32]. However, it was shown that the expression of *Il-18* gene can be upregulated following IFN priming via STAT1/2 and IRF9, although *Il-18* is constitutively expressed in BMDMs [31]. There is some evidence that the expression of caspase-11 can be induced by TLR4/TRIF-mediated activation of NF-κB pathway or by IFN-mediated activation of STAT1 [33]. Although the regulation of caspase-1 expression is poorly understood, IRF8 regulatory pathway was shown to have an impact on caspase-1 expression in B cells [34]. The expression of *Il-6* and *Tnfα* genes could be also regulated by the NF-κB pathway under the activation of TLRs (although it is likely that the NF-κB pathway does not play a major role in TNFα induction) [35,36]. Thus, based on our results, it is likely that OmpA could stimulate the NF-κB pathway in macrophages. It was already shown that *A. baumannii* OmpA can induce both the expression and the surface exposure of Toll-like receptor 2 in laryngeal epithelial cells [37]. Additionally, the effect of OmpA on the activation of BMDCs via TLR2, MAPK and NF-κB pathways was observed [38].

**Figure 3:**
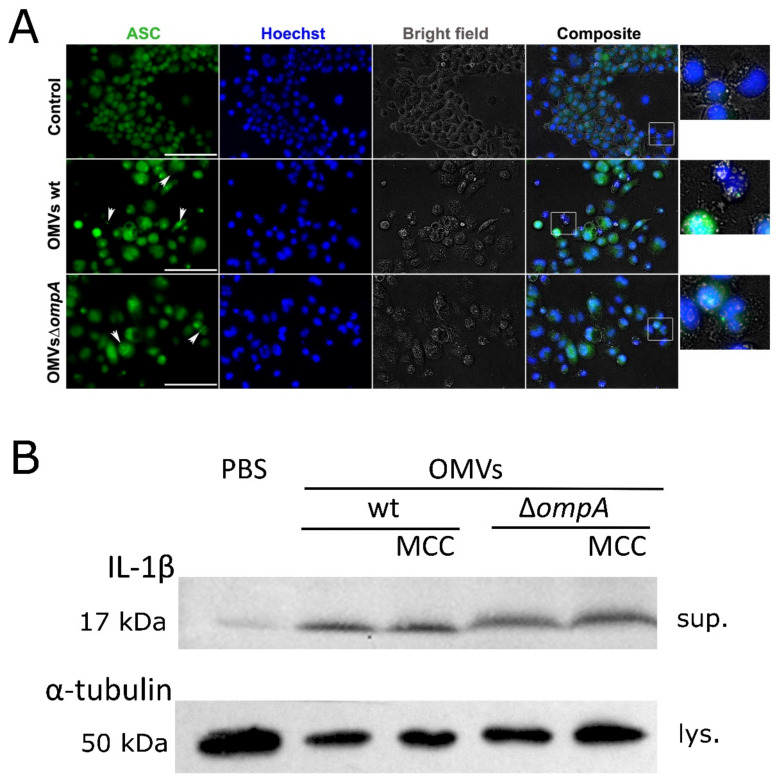
Inflamasomme induction in J774 macrophages stimulated with OMVs from *A. baumannii* wt and its ∆*ompA* mutant. (**A**) Immunofluorescence analysis of J774 mice macrophages incubated for 24 h with OMVs produced by *A. baumannii* wt strain and its *ompA* deletion mutant. Cells were stained with Hoechst and anti-ASC primary antibodies. ASC specks were visible as accumulated dots (indicated with white arrows). In the composite images, rectangles show magnified parts. Scale bar shows 100 µm; (**B**) detection of activated form of IL-1β (17 kDa) by Western blot. J774 macrophages were incubated with OMVs or PBS for 24 h. MCC—NLRP3 inflammasome inhibitor MCC950. Sup.—supernatants, lys.—cells lysates. Membrane was developed with Pierce ECL Western Blotting Substrate (Thermo Fisher Scientific, Walkersville, MD, USA).

The expression of *Il-1β* and *Il-18* genes results in the proactive cytokine forms, which should pass through the proteolytic maturation by caspase-1 and only then could be secreted outside the cell [30]. Therefore, we asked whether an inflammasome is activated in J774 macrophages when incubated with *A. baumannii* OMVs. The inflammasome formation was assessed by visualization of ASC specks using immunofluorescence analysis as described in Section 3. As can be seen in Figure 3A, ASC specks were observed as accumulated protein dots in macrophages, stimulated with OMVs obtained from both *A. baumannii* wt strain and its ∆*ompA* mutant.

Next, we investigated whether the pro-IL-1β cytokine activation occurs in J774 macrophages through the proteolytic cleavage. Western blot analysis with anti-IL-1β antibodies was performed on the supernatants of macrophages after their exposure to OMVs, since the active form of IL-1β is secreted into the extracellular space. As can be seen in Figure 3B, an IL-1β-specific band of 17 kDa, corresponding to the size of active protein form was observed in the fractions obtained from macrophages, stimulated with OMVs. A low level of activated cytokine is visible in the supernatant from macrophages incubated with PBS only, demonstrating basal level of activation in conditions tested. However, when macrophages were preincubated with NLRP3-inflammasome-specific inhibitor MCC950, the cleaved form of IL-1β was still found in the supernatants after the exposure to OMVs (Figure 3B). This observation suggests that IL-1β maturation could be triggered by the alternative inflammasomes or non-inflammasomal activation pathways. This is in agreement with the studies, where the ability of caspase-8 or other serine proteases to activate IL-1β in macrophages or dendritic cells has been demonstrated [29,39]. However, there are many ambiguities regarding the inflammation routes activated by *A. baumannii* infections. One of the recent studies has demonstrated that OmpA protein could act through the host GTPase dynamin-related protein 1 (DRP1) resulting in a profound mitochondrial fragmentation, elevation of the level of reactive oxygen species and finally cell death [9]. More investigations are needed to get a full picture of activated pathways during *A. baumannii*-induced inflammation.

Our results demonstrate the robust inflammatory properties of bacterial OMVs, what is consistent with other studies [40]. It is worth noting that OMVs produced by *A. baumannii* IC II (ST208) *ompA* deletion strain, similarly to its counterpart IC I (ST 203) strain, investigated in this study, activated reduced inflammatory response in J774 macrophages in vitro compared to wt strain (data not shown), thereby showing IC-common trait. Additionally, the *ompA* deletion in *A. baumannii* genome could result in some other changes in the composition of OMVs. This should be taken into account when considering the differences in the modulation of inflammatory response. *A. baumannii* OmpA-deficient OMVs still triggered acute proinflammatory response in macrophages, suggesting the impact of other components of OMVs in the inflammation. These components should be foreseen as possible targets for infection treatment, as it was already shown for OmpA [4]. By changing the composition of OMVs, bacteria could manipulate host immune system [41,42]. Therefore, a detailed examination of OMVs composition is needed considering the high diversity of virulence-related features of clinical *A. baumannii* strains [20,43].

## 3. Materials and Methods

### 3.1. Bacterial Growth Conditions and Manipulations

*A. baumannii* strain Ab_169_ and its *ompA* deletion mutant were described in a previous study [17]. OMVs were isolated as described by [17]. Briefly, 130 mL of bacterial culture in LB medium grown for 20 h at 37 °C was centrifuged for 15 min at 10,000× *g* at 4 °C. Supernatant was filtrated through a 0.22 µm filter. The OMVs were collected by ultracentrifugation at 130,000× *g* for 3 h at 4 °C. OMV pellets were resuspended in PBS. The protein concentration was determined using Bradford assay. Then, 10 µL of OMVs solution was plated on LB agar to test the sterility. Transmission electron microscopy (TEM) analysis was performed as described in previous study [17].

### 3.2. Cell Culture Assays

J774 mouse macrophages and A549 human lung epithelium cells were grown in Dulbecco’s modified Eagle’s medium (DMEM) supplemented with 10% of heat-inactivated FBS at 37 °C with 5% CO_2_. Cells were grown for 24 h to form a culture monolayer with ~80% confluence and were then exposed to OMVs.

The viability of eukaryotic cells was determined by using MTT substrate (Merck, Sigma-Aldrich, Darmstadt, Germany). Cells were incubated with 4 µg/mL of *A. baumannii* OMVs at 37 °C for 24 h. After incubation, the supernatant was aspirated and 0.2 mg/mL of MTT reagent was added to the cells and kept for 4 h at 37 °C. The supernatant was removed, and cells were incubated with 100 μL of isopropanol for 5 min. The absorbance at 570 nm was measured by Tecan Infinite M200 Pro plate reader (Tecan, Männedorf, Switzerland).

### 3.3. qPCR

J774 macrophages were incubated with 2 µg/mL OMVs at 37 °C at 5% CO_2_ for 24 h. After incubation, the supernatant was removed. The cells were carefully suspended in PBS buffer. The GeneJET RNA Purification Kit (Thermo Fisher Scientific) was used for RNA isolation according to the manufacturer’s recommendations. Genomic DNA was removed using deoxyribonuclease I (Thermo Fisher Scientific) following phenol/chloroform extraction. Concentration and purity were assessed with NanoDrop spectrophotometer (Thermo Fisher Scientific) and by agarose gel electrophoresis. cDNA synthesis was performed using the RevertAid First Strand cDNA Synthesis Kit (Thermo Fisher Scientific). Reaction components and conditions were selected according to the manufacturer’s recommendations. Gene expression was assessed by qPCR using DreamTaq polymerase (Thermo Fisher Scientific) and SYTO9 fluorescent dye (Thermo Fisher Scientific) according to the manufacturer’s recommendations. qPCR reactions were performed in CFX Real-Time System thermocycler (Bio-Rad, Hercules, CA, USA). The following primers used for qPCR were based on the previous studies [44,45,46]: actin—MuacF (5′-TGTCCACCTTCCAGCAGATGT-3′), MuacR (5′-TCAGTAACAGTCCGCCT-3′); TNFα—MutnF (5′-AGGAGAAAGTCAACCTCCT-3′), MutnR (5′-AAAGTAGACCTGCCCGGAC-3′); IL-6—Mu6F (5′-TCCAGTTGCCTTCTTGGGAC-3′), Mu6R (5′-GTACTCCAGAAGACCAGAGG-3′); caspase-1—mCaspase-1 RT fwd (5′-TTTCAGTAGCTCTGCGGTGT-3′), mCaspase-1 RT rev (5′-TTTCTTCCTGATTCAGCACTCTC-3′); caspase-11—mCaspase-11 RT fwd (5′-GCCACTTGCCAGGTCTACGAG-3′), mCaspase-11 RT rev (5′-AGGCCTGCACAATGATGACTTT-3′); NLRP3—mNLRP3 RT fwd (5′-CGAGACCTCTGGGAAAAAGCT-3′), mNLRP3 RT rev (5′-GCATACCATAGAGGAATGTGATGTACA-3′); IL-1β—IL-1betaF (5′-TGGACCTTCCAGGATGAGGACA-3′), IL-1betaR (5′-GTTCATCTCGGAGCCTGTAGTG-3′); IL-18—IL18F (5′-AGGACACTTTCTTGCTTGCC-3′), IL18R (5′-CACAAACCCTCCCCACCTAA-3′). The change in expression was compared to OMVs-untreated cells and was calculated by the ∆∆Ct method.

### 3.4. Immunoassays

For immunocytochemistry J774 macrophages were incubated with 1 µg/mL OMVs at 37 °C at 5% CO_2_ for 24 h. MCC950 inhibitor (InvivoGen, San Diego, CA, USA) was added into the wells 20 min before the exposure to OMVs to the final concentration of 1 μM. After the treatment cells were washed with PBS and fixed in 4% PFA dissolved in PBS for 15 min and permeabilized with 0.1% Triton X-100 prepared in PBS for 10 min. Cells were washed once and blocked with 2% BSA in PBS for 30 min. The primary antibodies rabbit anti-ASC (AB_2490440, AdipoGen, Liestal, Switzerland) at a dilution 1:200 were added to the blocking solution and incubated overnight. The following secondary antibody goat anti-rabbit (AB_142134, Thermo Fisher Scientific) was used at a dilution of 1:1000. The secondary antibody was applied for 2 h followed by two washing steps. Hoechst33342 was used for nuclear staining at 1 μg/mL for 30 min in PBS. Microscopy analysis was undertaken at the Department of Immunology and Cell Biology of Institute of Biotechnology (Vilnius University). ASC speck images were taken using a 40× objective. Photos were taken by EVOS FL Auto Imaging System (Thermo Fisher Scientific, USA). Acquired images were processed using ImageJ program (Wayne Rusband; National Institute of Health, Bethesda, MD, USA).

For Western blot analysis, proteins after separation by SDS-PAGE were transferred on nitrocellulose membrane (Amersham Biosciences, Pittsburgh, PA, USA) using the semi-dry method. Then, 1:5000 dilution of OmpA-specific serum obtained from immunized mice, 1:1000 dilution of goat anti-IL-1β (AF-401-SP, R&D Systems, Minneapolis, MN, USA) or 1:5000 dilution of rabbit anti-α tubulin (ab52866, Abcam, Cambridge, UK) antibodies were applied. Next, the membranes were exposed to anti-mouse IgG (H + L)-HRP conjugate (1706516, Bio-Rad, Marnes-la-Coquette, France), anti-goat IgG (H + L)-HRP conjugate (HAF017, R&D Systems, Minneapolis, MN, USA) or anti-rabbit IgG (H + L)-HRP conjugate (31460, Thermo Fisher Scientific, Walkersville, MD, USA) and developed with Pierce one-step ultra TMB blotting solution (Thermo Fisher Scientific, Walkersville, MD, USA) or Pierce ECL Western Blotting Substrate (Thermo Fisher Scientific, Walkersville, MD, USA).

### 3.5. Statistical Analysis

All statistical comparisons were based on t-test. All quantitative data are representative of at least three repeats.

## Data Availability

The data are available by request.

## Supplementary Materials

The following are available online at https://www.mdpi.com/article/10.3390/pathogens10040407/s1, Figure S1: Proteins of outer membrane vesicles (OMVs) from *A. baumannii* wt strain and its ∆*ompA* mutant.

## References

[1] Ayobami O., Willrich N., Harder T., Okeke I.N., Eckmanns T., Markwart R. (2019). The incidence and prevalence of hospital-acquired (carbapenem-resistant) *Acinetobacter baumannii* in Europe, Eastern Mediterranean and Africa: A systematic review and meta-analysis. Emerg. Microbes Infect..

[2] Morris F.C., Dexter C., Kostoulias X., Uddin M.I., Peleg A.Y. (2019). The Mechanisms of Disease Caused by *Acinetobacter baumannii*. Front. Microbiol..

[3] Iyer R., Moussa S.H., Durand-Réville T.F., Tommasi R., Miller A. (2018). *Acinetobacter baumannii* OmpA Is a Selective Antibiotic Permeant Porin. ACS Infect. Dis..

[4] Nie D., Hu Y., Chen Z., Li M., Hou Z., Luo X., Mao X., Xue X. (2020). Outer membrane protein A (OmpA) as a potential therapeutic target for *Acinetobacter baumannii* infection. J. Biomed. Sci..

[5] Choi C.H., Hyun S.H., Lee J.Y., Lee J.S., Lee Y.S., Kim S.A., Chae J.P., Yoo S.M., Lee J.C. (2008). *Acinetobacter baumannii* outer membrane protein A targets the nucleus and induces cytotoxicity. Cell Microbiol..

[6] Lee J.S., Choi C.H., Kim J.W., Lee J.C. (2010). *Acinetobacter baumannii* outer membrane protein A induces dendritic cell death through mitochondrial targeting. J. Microbiol..

[7] Jin J.S., Kwon S.O., Moon D.C., Gurung M., Lee J.H., Kim S.I., Lee J.C. (2011). *Acinetobacter baumannii* secretes cytotoxic outer membrane protein A via outer membrane vesicles. PLoS ONE.

[8] Kim S.Y., Kim M.H., Kim S.I., Son J.H., Kim S., Lee Y.C., Shin M., Oh M.H., Lee J.C. (2019). The sensor kinase BfmS controls production of outer membrane vesicles in *Acinetobacter baumannii*. BMC Microbiol..

[9] Tiku V., Kofoed E.M., Yan D., Kang J., Xu M., Reichelt M., Dikic I., Tan M.W. (2021). Outer membrane vesicles containing OmpA induce mitochondrial fragmentation to promote pathogenesis of *Acinetobacter baumannii*. Sci. Rep..

[10] Schwechheimer C., Kuehn M.J. (2015). Outer-membrane vesicles from Gram-negative bacteria: Biogenesis and functions. Nat. Rev. Microbiol..

[11] Kulp A., Kuehn M.J. (2010). Biological functions and biogenesis of secreted bacterial outer membrane vesicles. Annu Rev. Microbiol..

[12] Lin X.M., Yang J.N., Peng X.X., Li H. (2010). A novel negative regulation mechanism of bacterial outer membrane proteins in response to antibiotic resistance. J. Proteome Res..

[13] Manning A.J., Kuehn M.J. (2011). Contribution of bacterial outer membrane vesicles to innate bacterial defense. BMC Microbiol..

[14] Liu Y., Defourny K.A.Y., Smid E.J., Abee T. (2018). Gram-Positive Bacterial Extracellular Vesicles and Their Impact on Health and Disease. Front. Microbiol..

[15] Kim S.W., Park S.B., Im S.P., Lee J.S., Jung J.W., Gong T.W., Lazarte J.M.S., Kim J., Seo J.S., Kim J.H. (2018). Outer membrane vesicles from β-lactam-resistant *Escherichia coli* enable the survival of β-lactam-susceptible *E. coli* in the presence of β-lactam antibiotics. Sci. Rep.

[16] Jan A.T. (2017). Outer Membrane Vesicles (OMVs) of Gram-negative Bacteria: A Perspective Update. Front. Microbiol..

[17] Skerniškytė J., Karazijaitė E., Deschamps J., Krasauskas R., Briandet R., Sužiedėlienė E. (2019). The mutation of conservative asp268 residue in the peptidoglycan-associated domain of the ompa protein affects multiple *acinetobacter baumannii* virulence characteristics. Molecules.

[18] Marion C.R., Lee J., Sharma L., Park K.S., Lee C., Liu W., Liu P., Feng J., Gho Y.S., Dela Cruz C.S. (2019). Toll-like receptors 2 and 4 modulate pulmonary inflammation and host factors mediated by outer membrane vesicles derived from *acinetobacter baumannii*. Infect. Immun..

[19] Uppalapati S.R., Sett A., Pathania R. (2020). The outer membrane proteins ompa, caro, and oprd of *acinetobacter baumannii* confer a two-pronged defense in facilitating its success as a potent human pathogen. Front. Microbiol..

[20] Skerniškytė J., Krasauskas R., Péchoux C., Kulakauskas S., Armalytė J., Sužiedėlienė E. (2019). Surface-related features and virulence among *acinetobacter baumannii* clinical isolates belonging to international clones I and II. Front. Microbiol..

[21] Li Z.T., Zhang R.L., Bi X.G., Xu L., Fan M., Xie D., Xian Y., Wang Y., Li X.J., Wu Z.D. (2015). Outer membrane vesicles isolated from two clinical *Acinetobacter baumannii* strains exhibit different toxicity and proteome characteristics. Microb. Pathog..

[22] Smith S.G., Mahon V., Lambert M.A., Fagan R.P. (2007). A molecular Swiss army knife: OmpA structure, function and expression. FEMS Microbiol. Lett..

[23] Gaddy J.A., Tomaras A.P., Actis L.A. (2009). The *Acinetobacter baumannii* 19606 OmpA protein plays a role in biofilm formation on abiotic surfaces and in the interaction of this pathogen with eukaryotic cells. Infect. Immun..

[24] Smani Y., McConnell M.J., Pachón J. (2012). Role of fibronectin in the adhesion of *Acinetobacter baumannii* to host cells. PLoS ONE.

[25] Parameswaran N., Patial S. (2010). Tumor necrosis factor-α signaling in macrophages. Crit. Rev. Eukaryot. Gene Expr..

[26] Dikshit N., Kale S.D., Khameneh H.J., Balamuralidhar V., Tang C.Y., Kumar P., Lim T.P., Tan T.T., Kwa A.L., Mortellaro A. (2018). NLRP3 inflammasome pathway has a critical role in the host immunity against clinically relevant *Acinetobacter baumannii* pulmonary infection. Mucosal Immunol..

[27] Kang M.J., Jo S.G., Kim D.J., Park J.H. (2017). NLRP3 inflammasome mediates interleukin-1β production in immune cells in response to *Acinetobacter baumannii* and contributes to pulmonary inflammation in mice. Immunology.

[28] Man S.M., Karki R., Kanneganti T.D. (2017). Molecular mechanisms and functions of pyroptosis, inflammatory caspases and inflammasomes in infectious diseases. Immunol. Rev..

[29] Wan C.K., Li P., Spolski R., Oh J., Andraski A.B., Du N., Yu Z.X., Dillon C.P., Green D.R., Leonard W.J. (2015). IL-21-mediated non-canonical pathway for IL-1β production in conventional dendritic cells. Nat. Commun..

[30] Swanson K.V., Deng M., Ting J.P. (2019). The NLRP3 inflammasome: Molecular activation and regulation to therapeutics. Nat. Rev. Immunol..

[31] Zhu Q., Kanneganti T.D. (2017). Cutting Edge: Distinct Regulatory Mechanisms Control Proinflammatory Cytokines IL-18 and IL-1β. J. Immunol..

[32] Christgen S., Place D.E., Kanneganti T.D. (2020). Toward targeting inflammasomes: Insights into their regulation and activation. Cell Res..

[33] Schauvliege R., Vanrobaeys J., Schotte P., Beyaert R. (2002). Caspase-11 gene expression in response to lipopolysaccharide and interferon-gamma requires nuclear factor-kappa B and signal transducer and activator of transcription (STAT) 1. J. Biol Chem..

[34] Lv D.W., Zhang K., Li R. (2018). Interferon regulatory factor 8 regulates caspase-1 expression to facilitate Epstein-Barr virus reactivation in response to B cell receptor stimulation and chemical induction. PLoS Pathog..

[35] Tanaka T., Narazaki M., Kishimoto T. (2014). IL-6 in inflammation, immunity, and disease. Cold Spring Harb. Perspect. Biol..

[36] Falvo J.V., Tsytsykova A.V., Goldfeld A.E. (2010). Transcriptional control of the TNF gene. Curr. Dir. Autoimmun..

[37] Kim S.A., Yoo S.M., Hyun S.H., Choi C.H., Yang S.Y., Kim H.J., Jang B.C., Suh S.I., Lee J.C. (2008). Global gene expression patterns and induction of innate immune response in human laryngeal epithelial cells in response to *Acinetobacter baumannii* outer membrane protein A. FEMS Immunol. Med. Microbiol..

[38] Lee J.S., Lee J.C., Lee C.M., Jung I.D., Jeong Y.I., Seong E.Y., Chung H.Y., Park Y.M. (2007). Outer membrane protein a of *Acinetobacter baumannii* induces differentiation of CD4+ T cells toward a Th1 polarizing phenotype through the activation of dendritic cells. Biochem. Pharmacol..

[39] Chauhan D., Bartok E., Gaidt M.M., Bock F.J., Herrmann J., Seeger J.M., Broz P., Beckmann R., Kashkar H., Tait S.W.G. (2018). BAX/BAK-Induced Apoptosis Results in Caspase-8-Dependent IL-1β Maturation in Macrophages. Cell Rep..

[40] Kaparakis-Liaskos M., Ferrero R.L. (2015). Immune modulation by bacterial outer membrane vesicles. Nat. Rev. Immunol..

[41] Yun S.H., Park E.C., Lee S.Y., Lee H., Choi C.W., Yi Y.S., Ro H.J., Lee J.C., Jun S., Kim H.Y. (2018). Antibiotic treatment modulates protein components of cytotoxic outer membrane vesicles of multidrug-resistant clinical strain, *Acinetobacter baumannii* DU202. Clin. Proteomics..

[42] Nagakubo T., Nomura N., Toyofuku M. (2020). Cracking Open Bacterial Membrane Vesicles. Front. Microbiol..

[43] Loraine J., Heinz E., Soontarach R., Blackwell G.A., Stabler R.A., Voravuthikunchai S.P., Srimanote P., Kiratisin P., Thomson N.R., Taylor P.W. (2020). Genomic and Phenotypic Analyses of *Acinetobacter baumannii* Isolates From Three Tertiary Care Hospitals in Thailand. Front. Microbiol..

[44] Bouwman L.I., de Zoete M.R., Bleumink-Pluym N.M., Flavell R.A., van Putten J.P. (2014). Inflammasome activation by *Campylobacter jejuni*. J. Immunol..

[45] Huang Y.H., Molavi O., Alshareef A., Haque M., Wang Q., Chu M.P., Venner C.P., Sandhu I., Peters A.C., Lavasanifar A. (2018). Constitutive Activation of STAT3 in Myeloma Cells Cultured in a Three-Dimensional, Reconstructed Bone Marrow Model. Cancers.

[46] Yu Y., Fu S., Zhang X., Wang L., Zhao L., Wan W., Xue Y., Lv L. (2020). Leptin facilitates the differentiation of Th17 cells from MRL/Mp-Fas lpr lupus mice by activating NLRP3 inflammasome. Innate Immun..

