# Supplementary material for: OmpA Protein-Deficient Acinetobacter baumannii Outer Membrane Vesicles Trigger Reduced Inflammatory Response
Source: Pathogens. 2021 Mar 31;10(4):407. doi: 10.3390/pathogens10040407 (PMC8066360; doi:10.3390/pathogens10040407)
Supplement: Supplementary file 1 [file pathogens-10-00407-s001.pdf]

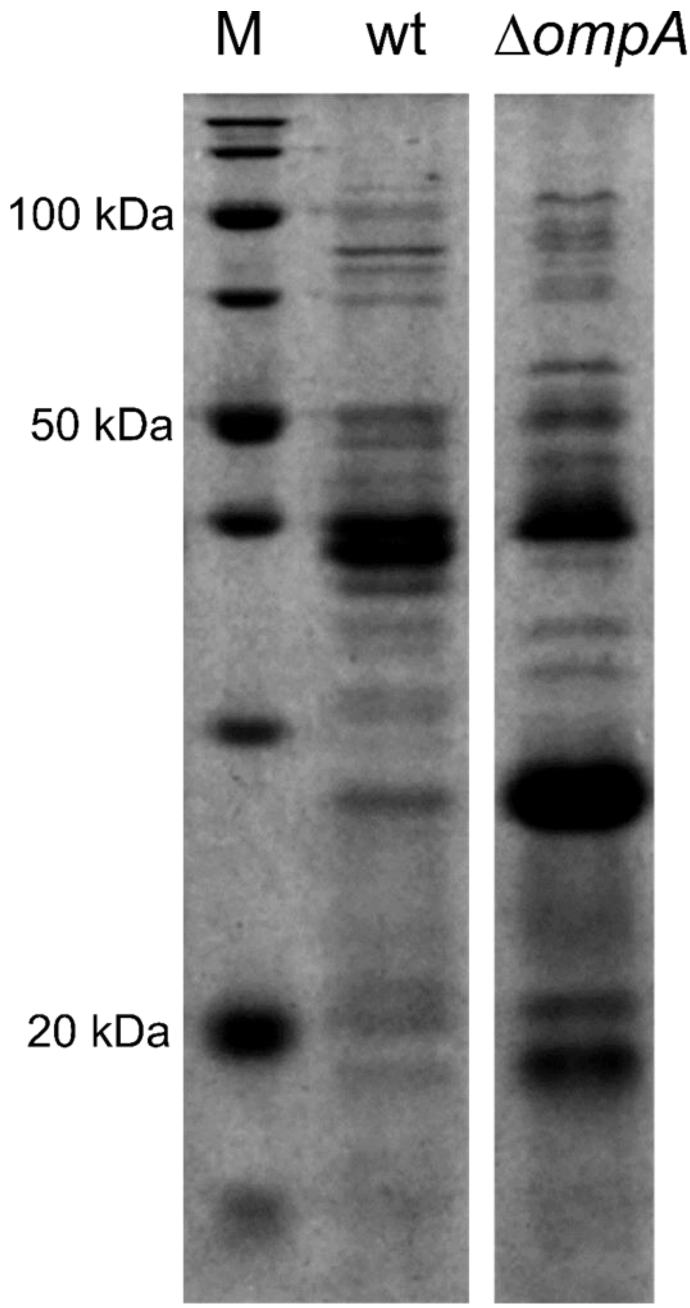

Figure S1. Proteins of outer membrane vesicles (OMVs) from *A. baumannii* wt strain and its Δ*ompA* mutant. Proteins were fractionated by SDS-PAGE and stained with Coomassie Brilliant Blue. M—PageRuler Unstained Broad Range Protein Ladder (Thermo Fisher Scientific).
